# Predicting Malignant Transformation of Choroidal Nevi Using Machine Learning

**DOI:** 10.21203/rs.3.rs-3778562/v1

**Published:** 2023-12-21

**Authors:** Sabrina P. Iddir, Jacob Love, Jiechao (Simon) Ma, John M. Bryan, Sanjay Ganesh, Michael J. Heiferman, Darvin Yi

**Affiliations:** Department of Ophthalmology and Visual Sciences, University of Illinois at Chicago; Department of Computer Science, University of Illinois at Chicago; Department of Biomedical Engineering, University of Illinois at Chicago; Department of Ophthalmology, Northwestern University; Department of Ophthalmology and Visual Sciences, University of Illinois at Chicago; Department of Ophthalmology and Visual Sciences, University of Illinois at Chicago; Department of Ophthalmology and Visual Sciences, University of Illinois at Chicago

**Keywords:** Uveal Melanoma, Choroidal Nevus, Malignant Transformation, Machine Learning, Artificial Intelligence

## Abstract

**Objective:**

This study aims to assess a machine learning (ML) algorithm using multimodal imaging to accurately identify risk factors for uveal melanoma (UM) and aid in the diagnosis of melanocytic choroidal tumors.

**Subjects and Methods:**

This study included 223 eyes from 221 patients with melanocytic choroidal lesions seen at the eye clinic of the University of Illinois at Chicago between 01/2010 and 07/2022. An ML algorithm was developed and trained on ultra-widefield fundus imaging and B-scan ultrasonography to detect risk factors of malignant transformation of choroidal lesions into UM. The risk factors were verified using all multimodal imaging available from the time of diagnosis. We also explore classification of lesions into UM and choroidal nevi using the ML algorithm.

**Results:**

The ML algorithm assessed features of ultra-widefield fundus imaging and B-scan ultrasonography to determine the presence of the following risk factors for malignant transformation: lesion thickness, subretinal fluid, orange pigment, proximity to optic nerve, ultrasound hollowness, and drusen. The algorithm also provided classification of lesions into UM and choroidal nevi. A total of 115 patients with choroidal nevi and 108 patients with UM were included. The mean lesion thickness for choroidal nevi was 1.6 mm and for UM was 5.9 mm. Eleven ML models were implemented and achieved high accuracy, with an area under the curve of 0.982 for thickness prediction and 0.964 for subretinal fluid prediction. Sensitivity/specificity values ranged from 0.900/0.818 to 1.000/0.727 for different features. The ML algorithm demonstrated high accuracy in identifying risk factors and differentiating lesions based on the analyzed imaging data.

**Conclusions:**

This study provides proof of concept that ML can accurately identify risk factors for malignant transformation in melanocytic choroidal tumors based on a single ultra-widefield fundus image or B-scan ultrasound at the time of initial presentation. By leveraging the efficiency and availability of ML, this study has the potential to provide a non-invasive tool that helps to prevent unnecessary treatment, improve our ability to predict malignant transformation, reduce the risk of metastasis, and potentially save patient lives.

## Introduction

Uveal melanoma (UM) is the most common intraocular malignancy in adults, with a high rate of metastasis and a poor prognosis.^1^ The accurate diagnosis of small UM is challenging due to similar clinical characteristics to benign choroidal nevi. Tumors diagnosed as choroidal nevi that subsequently grow during an observation period are at increased risk for metastasis.^2, 3^ Therefore, improving the diagnosis of UM and choroidal nevi at the time of initial presentation has the potential to improve clinical outcomes.

Most types of cancer require a tissue diagnosis via biopsy prior to making a treatment decision. However, the diagnosis of UM is usually made using a clinical diagnosis due to the morbidity associated with ocular biopsy.^4–6^ The risks associated with biopsy in small choroidal tumors are especially high.^7, 8^ Therefore, clinicians base their diagnosis on careful clinical examination and multimodal imaging, including fundus photography, autofluorescence, optical coherence tomography (OCT), and ultrasonography, to evaluate patients with melanocytic choroidal tumors. However, indeterminate lesions where a definite diagnosis cannot be made are often observed to monitor for tumor growth with serial examination and imaging.

Tumor growth is used as a surrogate for malignant transformation and, therefore, an indication for treatment in patients with indeterminate melanocytic choroidal tumors. Large retrospective studies have been performed to identify clinical risk factors that predict malignant transformation in order to identify patients at high risk for tumor growth. Patients at high risk for malignant transformation are most often treated with ionizing radiation or enucleation based on the clinical situation including tumor size, extent of extraocular extension, vision, and the patient’s preference. Conversely, patients at low risk for malignant transformation are often observed to avoid the unnecessary ocular morbidity associated with treatment of benign choroidal nevi.

The risk factors for malignant transformation have been well characterized and include tumor thickness greater than 2 mm, subretinal fluid, visual symptoms, orange pigment, proximity to the optic disc, ultrasonographic hollowness, and the absence of drusen.^9–12^ The presence of three or more of these features suggests a greater than 50% risk of malignant transformation.^13^ The use of multimodal imaging has been shown to be capable of identifying these risk factors and therefore serves as an important tool for clinicians evaluating these lesions^14^. However, improving our ability to predict malignant transformation and accurately diagnose small UM can reduce the risk of metastasis and save patient lives.

Machine learning (ML) offers a promising approach to enhance the identification and evaluation of intraocular lesions, thereby providing a versatile tool for clinicians. Deep learning, a subset of ML, has recently advanced many areas of computer vision, including image classification,^15–19^ object detection,^20–23^ and semantic segmentation.^24–29^ Convolutional neural networks (CNNs) assist in disease diagnostics and progress our understanding of the possibilities of extracted information from various imaging techniques. Despite its significant potential, few studies have looked at the role of ML in the diagnosis of melanocytic choroidal tumors.^30^ In the present study, we analyze the utility of ML in the evaluation of choroidal nevi and UM. Our objective was to train an ML algorithm to identify risk factors for UM using ultra-widefield fundus images and B-scan ultrasonography. In addition to providing useful information for the diagnosis of the disease itself, we also attempt to maximize the information we can extract from each imaging modality. As such, this ML algorithm may be a useful tool for evaluating melanocytic choroidal tumors for early detection of malignancy.

## Methods

This retrospective study included analysis of 223 eyes from 221 patients with melanocytic choroidal lesions seen at the eye clinic at the University of Illinois at Chicago between 01/2010 and 07/2022 (Table 1). The study was approved by the institutional review board (IRB) and patient records were collected from the electronic medical record system. The inclusion criteria for this study were patients with a clinical diagnosis of choroidal nevi or UM. Exclusion criteria were patients who have been treated prior to presentation and patients without both ultra-widefield imaging (Optos PLC, Dunfermline, Fife, Scotland, UK) and B-scan ultrasound (Eye Cubed and ABSolu, Lumibird Medical, Rennes, France) taken at the time of initial presentation. The patients were divided into two groups: (1) patients diagnosed with a choroidal nevus and (2) patients diagnosed with UM. The clinical examination and diagnosis at the time of presentation were taken as the ground truth for diagnosis and the presence of risk factors for malignant transformation included lesion thickness, subretinal fluid, orange pigment, proximity to optic nerve, ultrasound hollowness, and drusen. The risk factors were verified by a single investigator (MJH) using all multimodal imaging available from the time of diagnosis including ultra-widefield images (UWF), autofluorescence images, A-scan and B-scan ultrasonography (US), and OCT. We also explore prediction of the categorization into choroidal nevus or UM for each image. The UWF images and B-scan US from all patients were collected and analyzed (Table 2).

The AI-based models were developed using ResNet 18 architecture.^18^ TheResNet architecture consisted of two parts: (1) a feature extractor, which processed UWF or US images to extract features as an output, and (2) a task-specific header that used features from the previous layers to generate task-specific outputs (i.e., classification output 0 for absence of or apical overlying subretinal fluid, or output 1 for presence of subretinal fluid). Text information within the US images was cropped, and images were then scaled to 512 × 512 pixels before being fed into the models. Cross-entropy was used as the loss function, and the Adam algorithm was used for the optimizer. The learning rate was set to 0.00005 and the models were trained for 50 epochs. The best model was selected based on the lowest loss observed in the testing set.

The performance of the ML model was measured by the area under the curve (AUC). The bootstrap confidence interval (CI) for the AUC was obtained using the percentiles of the bootstrap distribution. For instance, the 95% CI was obtained using the 2.5th and 97.5th percentiles of the bootstrap distribution. The 95% CI was computed based on 1000 bootstrap replicates.

We investigated the region or tissue by generating saliency maps for visual explanations of each model using Gradient-Weighted Class Activation Mapping (Grad-CAM).^31^ Grad-CAM uses the gradients of the target concept, such as ‘UM’ in our classification network, flowing into the final convolutional layer. This produces a coarse localization map highlighting the important regions in the image for predicting the concept. The primary goal of Grad-CAM is to reflect the degree of importance of pixels (regions of interest) to the human visual system, allowing us to make decisions on the classification task.

## Results

### Patient demographics

A total of 115 patients with choroidal nevi and 108 patients with UM were included in this study. The mean age of patients with choroidal nevi was 64.9 years (range: 27–95), while patients with UM had a mean age of 66.1 years (range: 30–97) (Table 1). The majority of patients were female in the choroidal nevus group (75, 65.2%), and the UM group had a more balanced gender distribution with 53 (49.1%) males and 55 (50.1%) females. The racial distribution of patients in both groups was predominantly White, with 76 (73.8%) patients in the choroidal nevus group and 82 (83.7%) patients in the UM group among patients with available race data. Similarly, the ethnicity of patients in both groups was predominantly non-Hispanic or Latino, with 91 (85.8%) patients in the choroidal nevus group and 98 (96.1%) patients in the UM group.

### Clinical features

The mean lesion thickness was 1.6 mm for choroidal nevi and 5.9 mm for UM. The presence of subretinal fluid was observed in 5 (4.3%) patients with choroidal nevi and 75 (69.4%) patients with UM. Orange pigment was present in 3 (2.6%) patients with choroidal nevi and 34 (31.5%) patients with UM. The mean margin to the optic nerve head was 5.0 mm for choroidal nevi and 3.1 mm for UM. Drusen were present in 42 (36.5%) patients with choroidal nevi and 35 (32.4%) patients with UM. Ultrasonographic hollowness was observed in 18 (15.7%) patients with choroidal nevi and 86 (79.6%) patients with UM. Finally, a mushroom shape was not observed in any patients with choroidal nevi, while it was present in 16 (14.8%) patients with UM.

### AI-based models

**Figure 1:**
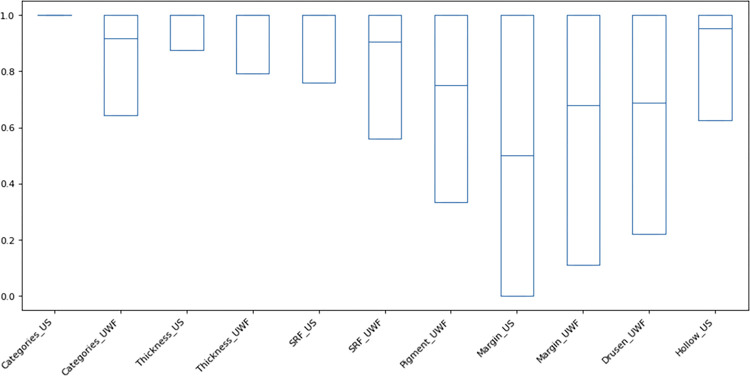
Boxplot of the bootstrap 95% confidence interval of the area under the curve (AUC). The vertical axis represents AUC values ranging from 0 to 1, with higher value indicating better predictive performance. The horizontal axis represents the different models trained and evaluated in this study. Each box indicates the interquartile range of the AUC distribution, with the horizontal line inside the box representing the median AUC value.

We trained 11 models in this study. The AI-based model achieved the following performance metrics: AUC of 0.982 (95% CI: 0.875–1), 0.964 (95% CI: 0.792–1) for thickness prediction with US or UWF, 0.963 (95% CI: 0.760–1) and 0.870 (95% CI: 0.560–1) for subretinal fluid prediction with US or UWF, 0.735 (95% CI: 0.333–1) for orange pigment prediction with UWF, 0.520 (95% CI: 0–1) and 0.667 (95% CI: 0.111–1) for margin prediction with US or UWF, 0.663 (95% CI: 0.222–1) for drusen prediction with UWF, 0.919 (95% CI: 0.625–1) for hollowness prediction with US, and 1 (95% CI: 1–1) and 0.894 (95% CI: 0.643–1) for category prediction with US or UWF, respectively (Fig. 1).

For models trained with US images, the sensitivity/specificity were as follows: 0.900/0.818 for thickness, 0.900/0.818 for subretinal fluid, 0.867/0.200 for margin to optic nerve head, 0.889/0.727 for ultrasonographic hollowness, and 0.818/1.000 for categories, respectively. For models trained with UWF images, the sensitivity/specificity were: 1.000/0.727 for thickness, 0.667/0.833 for subretinal fluid, 0/1 for orange pigment, 0.800/0.600 for margin, 0.375/0.846 for drusen, and 0.636/0.833 for categories, respectively. Grad-CAM images were generated to evaluate any localizing information.

### Grad-CAM images

**Figure 2:**
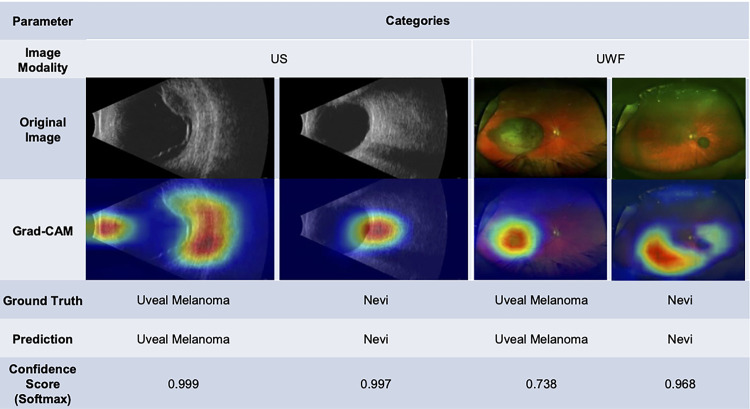
Representative ultrasound (US) and ultra-widefield fundus (UWF) original images, with a Grad-CAM saliency map showing the areas of highest probability for the category prediction model. Colors represent regions in order of decreasing performance: Red, Yellow, Green, Blue.

Localization maps highlight the important pixels (regions of interest) resulted in patterns that provided insight into the classification tasks. In the category prediction model from US images, the highest probability regions in the overlying Grad-CAM images tended to include both the lesion of interest and its surrounding tissues. For instance, the highlighted region of a UM included the orbit posterior to the tumor as well as ocular regions adjacent to the lesion (Fig. 2). Additionally, a subset of images highlighted the anterior segment on the US image in the location of the iris and lens, which have been implicated in patients with uveal melanoma.^32, 33^

From the UWF images, the Grad-CAM images most often correctly located the tumor region for UM. However, the localization maps for nevi tended to be broader and surrounded the lesions rather than focusing on the nevi themselves (Fig. 2). In one false negative case in the category prediction from US images (Supplemental Fig. 1, score of 0.289), the lesion is 1.94 mm in height but the largest basal diameter is 7.54 mm. As compared to other images of UM in the testing dataset, this image has the smallest thickness.

**Figure 3:**
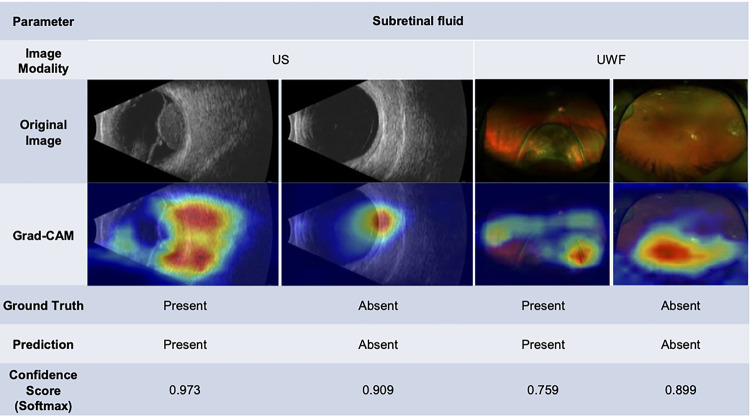
Ultrasound (US) and ultra-widefield fundus (UWF) original images, with a Grad-CAM saliency map showing the areas of highest probability for the subretinal fluid prediction model.

The subretinal fluid prediction model from US images often highlighted a two-centric region in Grad-CAM, corresponding to the subretinal fluid on two sides of the lesion with a confidence score of 0.973 (Fig. 3). The model was also capable of locating subretinal fluid from the UWF image with a confidence score of 0.759. Our model also consistently evaluated the predicted hollowness through visualization focusing more often on the lesion itself (Supplemental Fig. 2).

## Discussion

The accurate diagnosis of small melanocytic choroidal tumors is challenging due to similar clinical characteristics between benign choroidal nevi and small malignant UM. These patients benefit from the careful evaluation by an ocular oncologist experienced in managing intraocular tumors. Current practice uses clinical examination and multimodal imaging to predict malignant transformation and thereby guide the diagnosis and management of these tumors. Our study provides proof of concept for ML to identify risk factors for malignant transformation at the time of initial presentation.

Clinical features associated with the risk of malignancy have been well established, including the presence of orange pigment and subretinal fluid.^34, 35^ Shields et al. conducted a study to identify risk factors for malignant transformation of choroidal nevi, comprising the largest retrospective case series at the time.^11^ These risk factors included tumor thickness greater than 2 mm on ultrasonography, subretinal fluid, patient symptoms, orange pigment, and tumor margin within 3 mm of the optic disc.^11^ In 2009, Shields et al. expanded their case series and identified additional risk factors to include ultrasound hollowness and the absence of a halo or drusen overlying the lesion.^13^ They were combined to form the well-known mnemonic “To Find Small Ocular Melanoma Using Helpful Hints Daily” (TFSOM-UHHD).

While the original TFSOM system provided an evidence-based method for predicting malignant transformation of melanocytic choroidal tumors, Shields et al. further extended the system in 2019 with the development of the “To Find Small Ocular Melanoma Doing IMaging” (TFSOM-DIM) criteria.^14^ TFSOM-DIM incorporates multimodal imaging techniques in the identification of risk factors, including subretinal fluid on OCT, orange pigment on autofluorescence, and a basal diameter of at least 0.5 mm on fundus photography.^14^ These additional imaging techniques provided a more nuanced approach to identifying UM,^36^ which have been evaluated in subsequent studies. Geiger et al. used the TFSOM-DIM criteria to grade multimodal imaging by retrospective chart review, revealing significant differences in the range of risk scores between UM and choroidal nevi.^37^

Other groups have independently identified risk factors for malignant transformation in melanocytic choroidal tumors. The Collaborative Ocular Melanoma Study (COMS) analyzed small choroidal lesions to find that thickness greater than 2 mm, basal diameter greater than 12 mm, presence of orange pigment, and absence of drusen and RPE changes were predictive of tumor growth.^38^ Roelofs et al. developed a tumor categorization system, which provided a score for choroidal lesions based on five features: Mushroom shape, Orange pigment, Large size, Enlarging tumor, and Subretinal fluid.^39^ Their study found these criteria to have a sensitivity of 99.8% in identifying melanocytic choroidal tumors at risk for malignant transformation.^39^ These scoring systems emphasize the opportunity for objectivity in determining the distinction between the two lesions. Despite their potential in the prediction of malignancy, identifying these risk factors has traditionally been done through careful ophthalmic examination and image interpretation, which is subject to inter-observer variability.^40^ The application of ML algorithms, such as the one used in our study, has the potential to provide a more accurate and efficient system to improve patient prognosis.

The use of ML as a tool for evaluating retinal lesions has gained interest in recent years. ML involves training algorithms to learn from data sets to act on future data.^41^ While it has been shown to be useful in the early detection of diabetic retinopathy (DR),^42, 43^ its potential for predicting malignant transformation in UM has not yet been extensively explored.

In 2014, Roychowdhury et al. developed a novel, fully automated DR detection and grading system for automated screening and treatment prioritization, achieving a sensitivity of 100%, specificity of 53.26%, and an AUC of 0.904.^42^ In a study by Lam et al. (2018), the use of ML in DR was augmented by developing a CNN to recognize and distinguish between mild and multi-class DR on color fundus images with enhanced recognition of subtle characteristics.^43^

Supervised ML techniques have shown promise in classifying retinal disease type and stage.^44^ In the context of UM, wide-field digital true color fundus cameras can capture a choroidal nevus and its associated features in a single photo, potentially making data labeling and ML training faster and more efficient.^30^ This understanding suggests that ML may function as a valuable tool to assess small tumors and facilitate the prediction of malignant transformation.

Early detection and treatment of UM is crucial as metastasis events may occur early, and effective treatment can prevent its spread.^45, 46^ Despite the availability of effective treatments for the primary tumor, more than 50% of UM patients develop metastatic disease suggesting that UM may metastasize prior to the time of treatment.^45, 47^ Consequently, there is a need for identifying and treating small UM to minimize the number of melanocytic choroidal tumors that are observed and subsequently grow during the observation period. The importance of treating small UM was additionally emphasized by Eskelin et al., who measured doubling time in both untreated and treated metastatic UM and proposed that most metastases begin up to 5 years prior to primary tumor treatment.^48^ Murray et al. retrospectively evaluated a case series of small UM undergoing early fine-needle aspiration biopsy combined with pars plana vitrectomy and endolaser ablation.^47^ The study found no patients developed metastasis in the follow up period, suggesting that early treatment may lower the risk of mortality compared to observation alone.

Several studies have evaluated non-imaging biomarkers to better diagnose UM and predict prognosis, including some that employ ML techniques.^49–51^ Serum biomarkers, including several differentially expressed proteins identified in UM gene signatures, have been associated with a worse prognosis in patients diagnosed with UM.^49, 52, 53^ Furthermore, circulating tumor cells have been detected in patients without clinically detectable metastasis, indicating early spread and highlighting the need to identify prognostic biomarkers.^54, 55^ The search for these biomarkers underscores the importance of early detection of UM, potentially with the aid of ML for the diagnosis and management of UM. Small UM are a particularly important research subject due to the diagnostic challenge and potential for early local treatment to preserve vision and save lives.^56^

**Figure 4:**
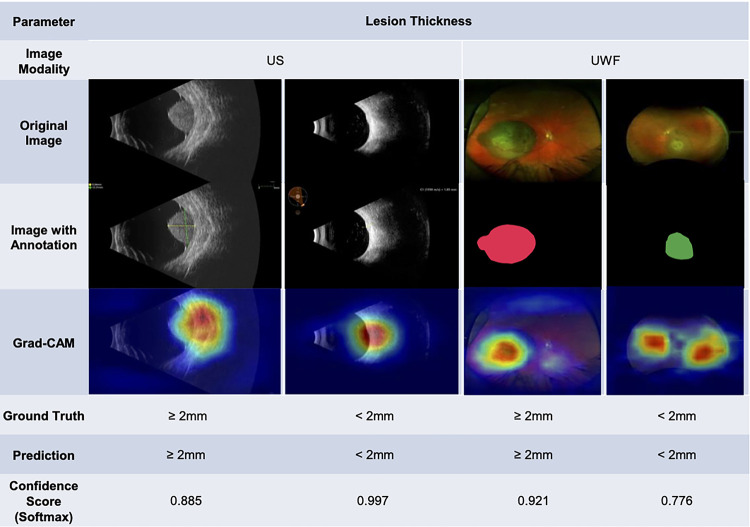
Representative ultrasound (US) and ultra-widefield fundus (UWF) original images, with a Grad-CAM saliency map showing the areas of highest probability for the thickness prediction model.

Our study has important limitations including a small sample size of patients from a single institution, which restricts the generalizability of our findings. Furthermore, the small sample size likely limited the performance of the ML models. The development of high-performing ML models to characterize choroidal lesions would benefit from multi-institutional collaborations and potentially techniques to artificially increase the sample size based on existing data. In addition, technical limitations such as poor image quality or suboptimal feature extraction can also limit the accuracy of ML models. In our study, the Grad-CAM images sometimes focused on small regions of the lesions themselves rather than adjacent subretinal fluid or whole image during the classification task (Fig. 3). To address this, segmentation of the specific regions based on the task, such as a lesion mask (Fig. 4) for orange pigment and drusen or the lesion plus the surrounding retina for subretinal fluid, could improve model performance. Our current classification model in margin, orange pigment, and drusen have relatively low average AUC and high deviation, suggesting the need for further refinement (Fig. 1). For the margin prediction model, segmentation of the optic nerve and lesion prior to training the model could be useful. In the case of orange pigment and drusen prediction models, the small size of these features may require alternative approaches such as cropping images into smaller tiles for classification, which could retain a higher resolution. Nonetheless, based on these limitations, expert knowledge is crucial to guide the development and use of these models to ensure that they are based on clinically relevant features and accurately reflect the underlying biology of the disease.

Our analysis provides proof of concept that ML can accurately identify risk factors for malignant transformation in melanocytic choroidal tumors based on a single UWF image or B-scan US image at the time of initial presentation. Further studies can build on these findings to improve the accuracy and applicability of these models in the clinical setting. ML has the potential to be developed into a clinically useful tool to inform and guide management decisions for melanocytic choroidal tumors and potentially save patient lives.

## Figures and Tables

**Table 1:** Clinical characteristics.

Parameter	Choroidal Nevus (n = 115)	Uveal Melanoma (n = 108)

Age (years)	Mean: 64.9	Mean: 66.1
	Range: 27–95	Range: 30–97

Sex (M/F)	40/75	53/55

Race	White: 76	White: 82
	Black or African American: 3	Black or African American: 1
	Asian: 1	Asian: 1
	Native Hawaiian/Pacific Islander: 0	Native Hawaiian/Pacific Islander: 0
	Other: 23	Other: 14
	No Information: 12	No Information: 10

Ethnicity	Hispanic or Latino: 15	Hispanic or Latino: 4
	Non-Hispanic or Latino: 91	Non-Hispanic or Latino: 98
	Unknown: 9	Unknown: 6

Lesion thickness (mm)	Mean: 1.6	Mean: 5.9

Subretinal fluid	5 (4.3%)	75 (69.4%)

Orange pigment	3 (2.6%)	34 (31.5%)

Margin to optic nerve head (mm)	Mean: 5.0	Mean: 3.1
	Range: 0–14	Range: 0–14

Drusen	42 (36.5%)	35 (32.4%)

Ultrasonographic hollowness	18 (15.7%)	86 (79.6%)

Mushroom shape	0 (0.0%)	16 (14.8%)

**Table 2:** Class descriptions and imaging modalities used for each parameter. US = ultrasound, UWF = ultra-widefield fundus.

Parameter	Class 0	Class 1	Imaging Modality
Lesion thickness (mm)	Smaller than 2mm	Greater than or equal to 2mm	US, UWF
Subretinal fluid	Absence or apical	Presence	US, UWF
Orange pigment	Absence or indeterminate	Presence or trace	UWF
Margin to optic nerve head (mm)	Greater than or equal to 3mm	Smaller than 3mm	US, UWF
Ultrasonographic hollowness	Absence or indeterminate	Presence	US
Drusen	Absence or indeterminate	Presence	UWF
Categories	Choroidal nevus	UM	US, UWF
